# Extracellular microRNA 130b‐3p inhibits eCIRP‐induced inflammation

**DOI:** 10.15252/embr.201948075

**Published:** 2019-11-14

**Authors:** Steven D Gurien, Monowar Aziz, Hui Jin, Haichao Wang, Mingzhu He, Yousef Al‐Abed, Jeffrey M Nicastro, Gene F Coppa, Ping Wang

**Affiliations:** ^1^ Center for Immunology and Inflammation The Feinstein Institutes for Medical Research Manhasset NY USA; ^2^ Department of Surgery Donald and Barbara Zucker School of Medicine at Hofstra/Northwell Manhasset NY USA; ^3^ Center for Biomedical Science The Feinstein Institutes for Medical Research Manhasset NY USA; ^4^ Center for Molecular Innovation The Feinstein Institutes for Medical Research Manhasset NY USA

**Keywords:** eCIRP, inflammation, macrophages, miRNA 130b‐3p, sepsis, Immunology, RNA Biology

## Abstract

Although microRNAs regulate mRNA expression intracellularly, they are often released into the circulation in inflammatory diseases. During sepsis, secreted extracellular cold‐inducible RNA‐binding protein (eCIRP) acts as a damage‐associated molecular pattern (DAMP), inducing tissue damage by elevating inflammatory cytokines and chemokines. Here, we report that the circulating microRNA 130b‐3p inhibits eCIRP‐mediated sterile and cecal ligation and puncture (CLP)‐induced non‐sterile inflammation. We find that levels of miR‐130b‐3p are increased in the serum of septic mice and patients and that it strongly interacts with recombinant murine (rm) CIRP *in vitro* and with eCIRP in the serum of septic mice *in vivo*. Combining a miR‐130b‐3p mimic with rmCIRP significantly decreases TNF‐α release by macrophages compared to only rmCIRP‐treated cells. This combined treatment also dose‐dependently decreases the affinity of rmCIRP with its receptor TLR4/MD2. Finally, injection of a miR‐130b‐3p mimic significantly reduces rmCIRP‐ or CLP‐induced systemic inflammation and acute lung injury in mice. These data show that extracellular miR‐130b‐3p functions as a novel endogenous inhibitor of eCIRP and point to an innovative therapeutic approach to treat inflammatory diseases.

## Graphical abstract

The DAMP eCIRP is secreted during sepsis and induces pro‐inflammatory cytokine production in macrophages. Extracellular miR‐130b‐3p binds to eCIRP and inhibits its interaction with its receptor, subsequently inhibiting pro‐inflammatory cytokine production and sepsis.

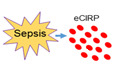

## Introduction

Inflammation occurs from the activation of the immune cells particularly macrophages by pathogen‐associated molecular patterns (PAMPs) or damage‐associated molecular patterns (DAMPs) 1. Sepsis, trauma, hemorrhage, and ischemia–reperfusion (I/R) lead to the release of DAMPs and subsequently cause inflammation 1, 2. DAMPs include but are not limited to high‐mobility group box 1 (HMGB1), mitochondrial DNA, and cellular chaperones (e.g., heat shock proteins) 3. Toll‐like receptors (TLRs) are located both intracellularly and on the cell surface of macrophages. These receptors respond to several different PAMPs and DAMPs 3, 4, 5, and transduce an intracellular signal, leading to the activation of nuclear factor‐κB (NF‐κB) and other inflammatory pathways 5.

Cold‐inducible RNA‐binding protein (CIRP) is a small 172‐amino‐acid RNA chaperone protein 6 and is secreted into the extracellular space in inflammatory diseases such as sepsis, I/R injuries, and hemorrhagic shock (reviewed in 7). Extracellular CIRP (eCIRP) acts as a DAMP which causes inflammation and tissue damage via elevating inflammatory cytokines and chemokines 7. eCIRP accomplishes this by binding to the TLR4 and myeloid differentiation factor 2 (MD2) complex on macrophages, leading to the activation of NF‐κB 8, 9. This causes the downstream effect of elevated levels of tumor necrosis factor‐α (TNF‐α), interleukin‐6 (IL‐6), HMGB1, and IL‐1β 7, 8, 9.

MicroRNAs (miRNAs) are short RNA molecules approximately 22 base pairs long 10. They are transcribed in the nucleus as primary miRNAs. After several modifications occurring in both the nucleus and the cytoplasm, primary miRNAs become double‐stranded miRNAs. The lead strand is then loaded onto an Argonaute protein to form an RNA‐induced silencing complex (RISC), while the passenger strand is lost 10, 11. RISC then binds to and inhibits the translation of specific mRNAs, usually by making them unstable and creating a shorter half‐life. Since binding does not require perfect complimentary between the mRNA and the miRNA, each miRNA may bind several hundred different types of mRNAs 12.

While the physiological role of miRNAs has mostly been found to occur intracellularly, miRNAs have also been identified in the extracellular space 13. They are surprisingly stable in the blood and can be measured in plasma 14, 15. This has prompted many studies to look into the efficacy of using extracellular miRNAs as diagnostic and prognostic markers for different diseases such as sepsis, cancers, and autoimmune diseases 16, 17, 18. The precise role of extracellular miRNA has yet to be adequately understood. A recent study demonstrated that treatment of mice and macrophages with selected extracellular miRNA mimics induced the expression of pro‐inflammatory cytokines through a TLR7/MyD88‐dependent pathway following their transfection into the cells 19. During sepsis, eCIRP and certain extracellular miRNA levels both become elevated in the blood 8, 17. While it has been shown that RNA‐binding protein CIRP interacts with nascent mRNA 6, 7, 20, the interaction between eCIRP and extracellular miRNA, and the impact of this extracellular interaction on immune cells has never been studied before. We therefore hypothesized that certain miRNAs released into the circulation can interact with eCIRP and regulate eCIRP's binding to TLR4. Out of the three miRNAs 130b‐3p, 27b, and 140, we found that miRNA 130b‐3p had the highest elevation in the serum during sepsis, the strongest binding affinity to eCIRP, and the highest inhibition of eCIRP‐induced inflammation via macrophages. Extracellular miRNA 130b‐3p serves as a novel endogenous inhibitor of eCIRP, and thus potentially is a novel therapeutic tool in counteracting inflammation.

## Results

### Sepsis increases serum levels of miRNA 130b‐3p

**Figure 1:**
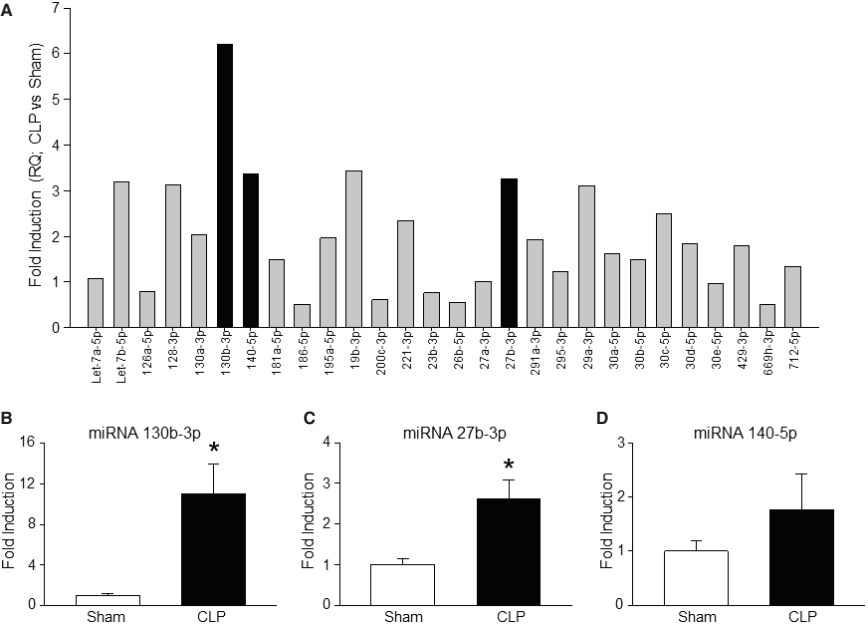
MicroRNA 130b‐3p is increased in the serum during sepsis Mice either underwent CLP for 24 h, or underwent laparotomy alone with no CLP (sham mice). Blood samples were collected at 24 h and miRNA was extracted, which was then used for PCR array profiling using qRT–PCR. 
ABar diagram representing the fold increase in the levels of serum miRNAs of CLP vs. sham mouse obtained from inflammatory pathway‐specific PCR array analysis (*n* = 1 mouse/group) is shown. The cycle threshold (CT) values of serum miRNAs of sham and CLP mice are presented in Fig EV1. *Caenorhabditis elegans* miRNA 39‐3p was spiked in and used to normalize comparative CT values (Table EV1). Relative quantities (fold induction) compared to sham mouse serum miRNAs were determined by using 2^−ddCT^.B–DValidation of increased circulating (B) miRNA 130b‐3p (*n* = 8 mice/group), (C) miRNA 27b (*n* = 8 mice/group), and (D) miRNA 140 (*n* = 8 mice/group). *C. elegans* miRNA 39‐3p was spiked in and used to normalize comparative CT values (Table EV2). Relative quantities (fold induction) compared to sham mouse serum miRNAs were determined by using 2^−ddCT^.Data information: Data are expressed as means ± SE and compared by Student's *t*‐test. *P <* *0.05 vs. sham. CLP, cecal ligation and puncture.

**Figure EV1:**
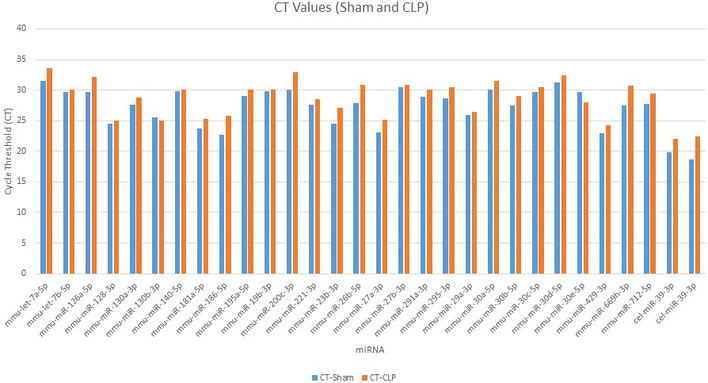
Cycle threshold (CT) of miRNAs in the serum of sham and septic mice After 24 h of sham or CLP operation in mice, serum samples were collected and miRNAs were extracted. PCR array was performed in an Applied Biosystems StepOnePlus real‐time PCR machine under the thermal profile of 95°C for 10 min and followed by 33 cycles of 95°C for 15 s and 60°C for 1 min. *Caenorhabditis elegans* miRNA 39‐3p was spiked in and used to normalize comparative cycle threshold (CT) values.

Our PCR array data revealed that several miRNAs linked to inflammatory pathways were increased in the serum of septic mice (Figs 1A and EV1, Table EV1). We found that miRNAs 130b‐3p, 27b, and 140, which have all been highly implicated in inflammation 21, 22, 23, were markedly upregulated in the serum of septic mice (Fig 1A). Further confirmation by RT–qPCR in the upregulation of serum levels of miRNAs 130b‐3p, 27b, and 140 of septic mice revealed that miRNA 130b‐3p was significantly increased by 11 ± 3.2‐fold compared to sham mice. By contrast, miRNA 27b and miRNA 140 had a fold increase in their serum levels by only 2.6 ± 0.4 and 1.8 ± 0.6, respectively (Fig 1B–D; Table EV2). These data suggest that sepsis leads to an efflux of these three miRNAs into the circulation during sepsis, with miRNA 130b‐3p having the greatest increase.

### Serum levels of miRNA 130b‐3p and eCIRP are both increased in septic patients

**Figure 2:**
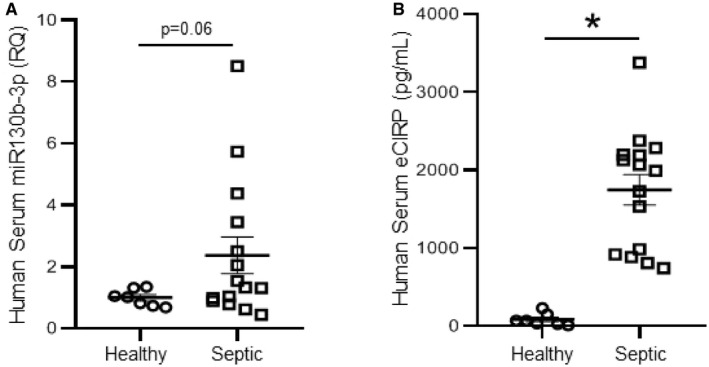
Increased levels of miRNA 130b‐3p and eCIRP in the serum of human septic patients Total microRNA was extracted from the blood samples of healthy individuals and septic patients.
miRNA 130b‐3p expression in their serum samples was determined by qRT–PCR and represented in scatter plot. *Caenorhabditis elegans* miRNA 39‐3p was spiked in and used to normalize comparative CT values (Table EV3). Relative quantities (fold induction) compared to healthy individuals’ serum miRNAs were determined by using 2^−ddCT^.eCIRP levels were quantified in the serum samples of healthy controls and septic patients.Data information: Data are expressed as means ± SE and compared by Student's *t*‐test (*n* = 7 healthy control and 15 septic patients). *P <* *0.05 vs. healthy control. eCIRP, extracellular cold‐inducible RNA‐binding protein.

Since our murine model of sepsis showed significantly increased levels of miRNA 130b‐3p in the serum compared to sham mice (Fig 1A and B), we next aimed to determine the serum levels of miR130b‐3p in healthy human subjects and septic patients. In order to do this study, we obtained de‐identified serum samples from 15 septic patients and seven healthy individuals. Patient samples were obtained from the Department of Emergency Medicine, North Shore University Hospital, Manhasset, New York. We found that the levels of miR130b‐3p in the serum of septic patients were markedly elevated by 2.4‐fold compared to healthy individuals (Fig 2A, Table EV3). We assessed eCIRP levels in the serum of these patients and healthy controls and found that septic patients had significantly higher levels of eCIRP in their blood compared to healthy individuals (Fig 2B).

### miRNA 130b‐3p has a strong binding affinity to CIRP and attenuates eCIRP's induction of pro‐inflammatory cytokines in macrophages

**Figure 3:**
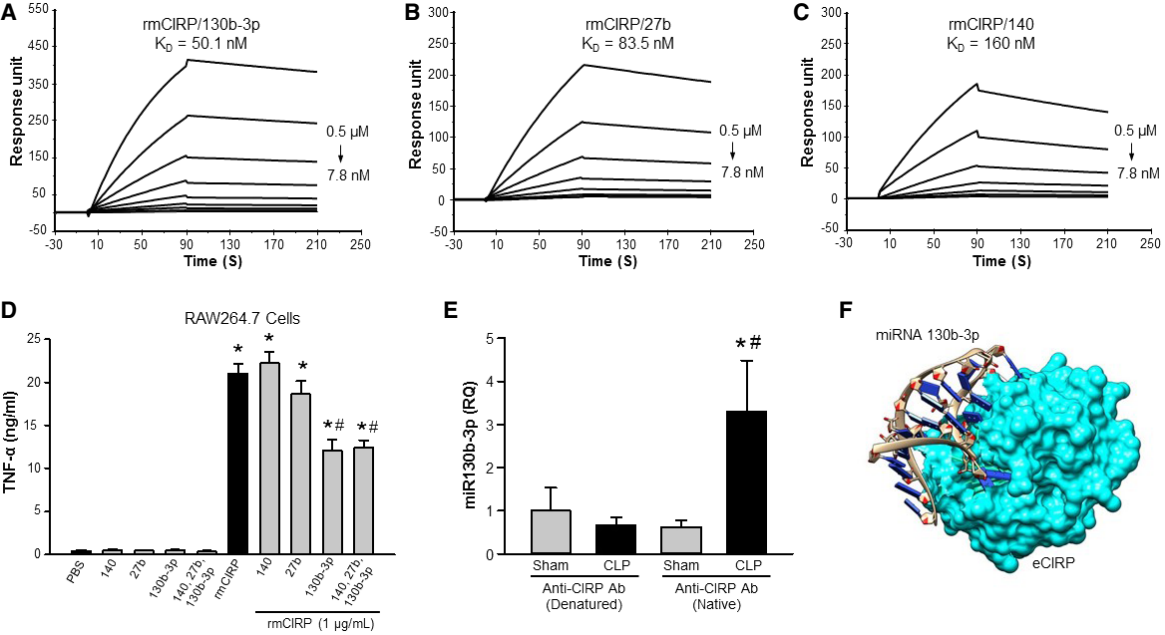
MicroRNA 130b‐3p exhibits the strongest binding affinity with rmCIRP and the highest TNF‐α inhibition on RAW264.7 cells A–CBiotinylated single‐stranded forms of miRNAs 130b‐3p, 27b, and 140 were individually immobilized on a sensor chip SA, and rmCIRP of varying concentrations was injected as the analyte. rmCIRP binds to (A) miRNA 130b‐3p mimic with a *K*_d_ of 50.1 ± 3.0 nM; (B) miRNA 27b mimic with a *K*_d_ of 83.5 ± 3.1 nM; and (C) miRNA 140 mimic with a *K*_d_ of 160 ± 1.9 nM. At least three (*n* = 3) independent BIAcore experiments were performed.DRAW264.7 cells (1 × 10^6 ^cells/ml) were stimulated with rmCIRP (1 μg/ml) with and without each of the three miRNAs at a concentration of 100 nM or altogether with each at a concentration of 100 nM. The supernatant was collected at 24 h and assessed for TNF‐α by ELISA.E, FeCIRP binds to extracellular miRNA 130b‐3p *in vivo*. Serum was isolated 20 h after CLP, or sham operation was performed on mice. Immunoprecipitation for eCIRP was then carried out on the serum by reacting the serum with either native CIRP Ab or denatured CIRP Ab. (E) All miRNAs were isolated from this IP, and miRNA 130b‐3p was amplified with subsequent quantification using qPCR and compared to denatured CIRP Ab control (*n* = 6 mice/group). *Caenorhabditis elegans* miRNA 39‐3p was spiked in and used to normalize comparative CT values. Relative quantities (fold induction) compared to sham mouse serum miRNAs treated with denatured CIRP Ab were determined by using 2^−ddCT^. (F) Virtual modeling illustrating docking of miRNA 130b‐3p and eCIRP. rmCIRP, recombinant murine cold‐inducible RNA‐binding protein.Data information: Data are expressed as means ± SE and compared by one‐way ANOVA and SNK method (*n* = 6 samples/group). (D) *P <* *0.001 vs. PBS, ^#^ *P *<* *0.05 vs. rmCIRP. (E) *P <* *0.05 vs. sham serum treated with denatured CIRP Ab and ^#^ *P *<* *0.05 vs. sham serum treated with native CIRP Ab.

**Figure EV2:**
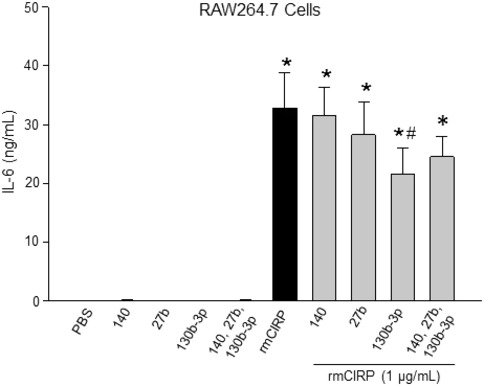
MicroRNA 130b‐3p attenuates eCIRP‐induced IL‐6 production in RAW264.7 cells *in vitro* RAW264.7 cells (1 × 10^6^ cells/ml) were stimulated with rmCIRP (1 μg/ml) with and without each of the three miRNAs at a concentration of 100 nM or altogether with each at a concentration of 100 nM. The supernatant was collected at 24 h and assessed for IL‐6 by ELISA. Data are expressed as means ± SE and compared by one‐way ANOVA and SNK method (*n* = 6 samples/group). *P <* *0.001 vs. PBS. ^#^ *P *< 0.05 vs. rmCIRP.

Cold‐inducible RNA‐binding protein has an N‐terminal RNA‐binding domain 7, 20. However, whether or not eCIRP binds to miRNAs is unknown. To determine the possible binding of miRNAs to eCIRP, a BIAcore study was performed on the three miRNAs 130b‐3p, 27b, and 140. We found that miRNA 130b‐3p had the strongest binding affinity to rmCIRP with a *K*_d_ value of 5.0 × 10^−8 ^M (Fig 3A). Although miRNA 27b and miRNA 140 had strong binding affinities to rmCIRP with *K*_d_ values of 8.4 × 10^8^ and 16 × 10^−8 ^M, respectively, they were 1.7 and 3.2 times weaker than miRNA 130b‐3p's binding to rmCIRP (Fig 3B and C). Since rmCIRP binds to miRNAs 130b‐3p, 27b, and 140, we therefore hypothesized that binding of rmCIRP with these miRNAs’ mimics would alter rmCIRP's functional effects. We looked at TNF‐α production of RAW264.7 macrophages when rmCIRP alone was added, and when rmCIRP was combined with one of the three miRNAs’ mimics before being added to the cells. Treatment of RAW264.7 cells with rmCIRP alone dramatically increased TNF‐α release (Fig 3D). Co‐treatment of cells with rmCIRP and miRNA 140 mimic did not exert any inhibitory effect on TNF‐α production, while miRNA 27b only had a mild inhibition (Fig 3D). Surprisingly, miRNA 130b‐3p mimic displayed a significant inhibitory effect of rmCIRP‐induced TNF‐α release by 42% (Fig 3D). Since miRNAs 130b‐3p, 140, and 27b are all recognized by eCIRP (Fig 3A–C), we next wanted to reveal whether or not the addition of miRNA 140 and 27b mimics along with miRNA 130b‐3p mimic can enhance or attenuate the inhibitory effects miRNA 130b‐3p mimic has on rmCIRP‐mediated TNF‐α production by macrophages. We found that there was no additional inhibition of rmCIRP‐induced TNF‐α or IL‐6 production by the macrophages following treatment of cells with miR130b, 140, and 27b altogether compared to condition where only miR130b‐3p mimic was added (Figs 3D and EV2). This implicated miRNA 130b‐3p in having a functional role of inhibiting TNF‐α production by macrophages after interacting with rmCIRP.

### Extracellular miRNA 130b‐3p binds to eCIRP *in vivo*

To determine whether extracellular miRNA 130b‐3p and eCIRP interact *in vivo*, a co‐immunoprecipitation (co‐IP) experiment was carried out on the serum of sham and septic mice 20 h after CLP was performed. Either native form of CIRP Ab or denatured CIRP Ab as negative control was incubated with the serum before a magnetic co‐IP was carried out. At the end of the co‐IP, miRNA attached to the eCIRP was isolated and miRNA 130b‐3p was quantified by real‐time qPCR. As shown in Fig 3E, the RQ of eCIRP‐bound miRNA 130b‐3p of the serum of sham and CLP mice following *ex vivo* treatment with denatured CIRP Ab was low and did not differ from each other. Surprisingly, the serum of CLP mice following *ex vivo* treatment with native CIRP Ab showed significant increase in the RQ of eCIRP‐bound miRNA 130b‐3p compared to sham mouse serum as well as to the serum of sham and CLP mice treated with denatured CIRP Ab (Fig 3E). These data clearly demonstrate eCIRP's binding to extracellular miRNA 130b‐3p under *in vivo* condition and also suggest that the content of eCIRP‐bound extracellular miRNA 130b‐3p was dramatically increased during sepsis. Besides these *in vivo* findings, computational analysis illustrated the theoretical modeling of the two molecules docking (Fig 3F).

### Dose response of miRNA 130b‐3p's inhibition of eCIRP's pro‐inflammation

**Figure 4:**
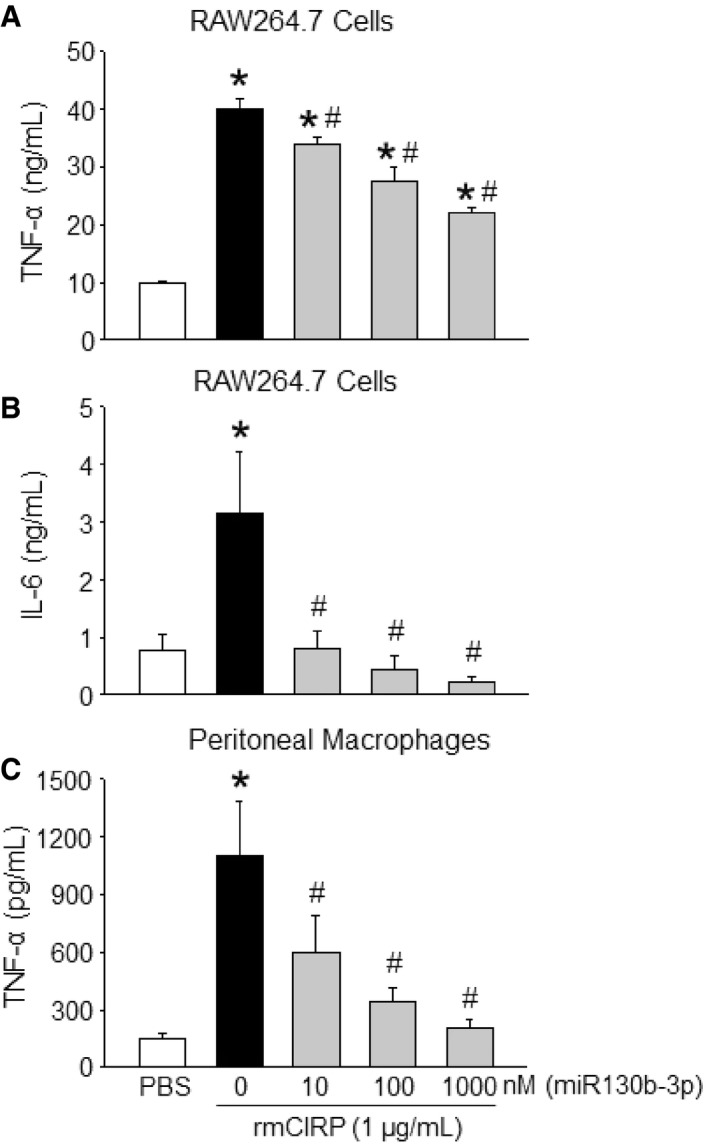
MicroRNA 130b‐3p attenuates eCIRP‐induced pro‐inflammatory responses in macrophages *in vitro* A, BRAW264.7 cells (1 × 10^6^ cells/ml) were treated with PBS or rmCIRP (1 μg/ml) with various doses (10, 100, and 1,000 nM) of miRNA 130b‐3p mimic. After 24 h of stimulation, supernatants were collected and assessed for TNF‐α and IL‐6 by ELISA.CA total of 1 × 10^6^ cells/ml of peritoneal macrophages were treated with PBS or rmCIRP (1 μg/ml) with various doses (10, 100, and 1,000 nM) of miRNA 130b‐3p mimic. After 24 h of stimulation, supernatants were collected and assessed for TNF‐α by ELISA. rmCIRP and miRNAs mimics were combined 30 min prior to stimulation to macrophage cells.Data information: Data are expressed as means ± SE (*n* = 4–6 samples in each group) and compared by using one‐way ANOVA and SNK method (*P <* *0.05 vs. PBS; ^#^ *P *<* *0.05 vs. rmCIRP treatment).

**Figure EV3:**
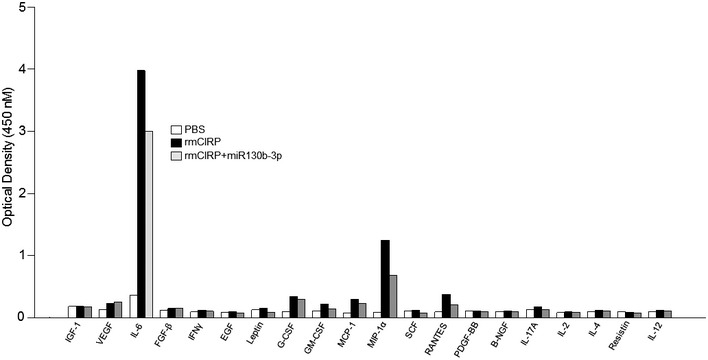
Effect of miRNA 130b‐3p mimic on cytokine production in rmCIRP‐treated peritoneal macrophages *in vitro* A total of 1 × 10^6^ cells/ml of peritoneal macrophages were treated with PBS or rmCIRP (1 μg/ml) with 100 nM of miRNA 130b‐3p mimic. After 24 h of stimulation, supernatants were collected and assessed for various cytokines using Mouse Cytokine ELISA Plate Array I. rmCIRP and miRNA 130b‐3p mimic were combined 30 min prior to stimulation to macrophage cells. Supernatants from *n* = 3 wells/group were pooled and performed for ELISA array. Data are expressed as optical density at 450 nM.

After increasing the concentration of miRNA 130b‐3p from 10 to 1,000 nM, there was a dramatic reduction in the expression of TNF‐α and IL‐6 at their protein levels by 45 and 81%, respectively, in the culture supernatants compared to rmCIRP‐treated RAW264.7 cells alone (Fig 4A and B). Similarly, with primary murine peritoneal macrophages, treatment of rmCIRP with 1,000 nM of miRNA 130b‐3p mimic significantly inhibited rmCIRP alone effects on the protein production of TNF‐α in the culture supernatants by 92% (Fig 4C). We extended the list of the inflammatory readouts by performing a multiplex ELISA array on the culture supernatants of the primary peritoneal macrophages treated with rmCIRP in the presence or absence of miR130b‐3p mimic. We found that miRNA 130b‐3p mimic attenuated the release of a wide range of inflammatory and pleiotropic cytokines including IL‐6, G‐CSF, GM‐CSF, MCP‐1, MIP‐1α, RANTES, and IL‐17A by the rmCIRP‐treated macrophages (Fig EV3). Thus, miRNA 130b‐3p attenuates eCIRP's pro‐inflammatory effects on murine macrophages.

### miRNA 130b‐3p mimic protects animals from rmCIRP‐induced systemic inflammation, lung inflammation, and acute lung injury

**Figure 5:**
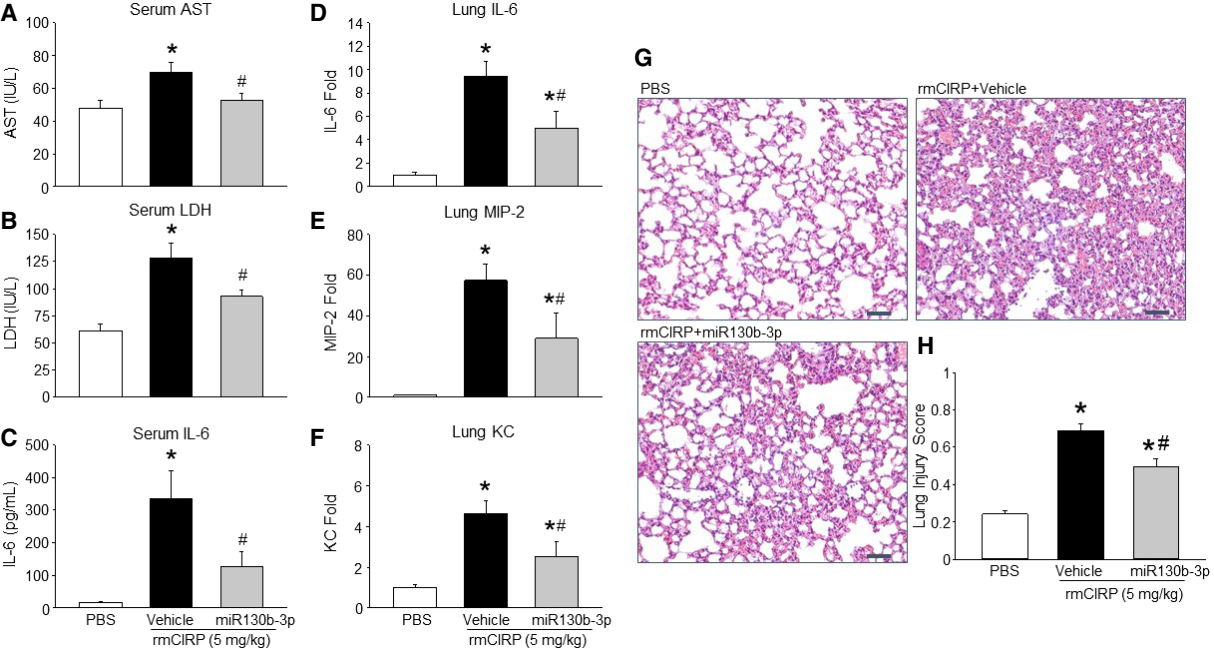
Treatment of mice with miRNA 130b‐3p mimic attenuates eCIRP‐induced pro‐inflammatory responses and improves overall lung histology Mice were injected with PBS or rmCIRP (5 mg/kg BW) *i.v*. together with or without miRNA 130b‐3p at a dose of 12.5 μl of 1,000 μM. In the miRNA 130b‐3p treatment group, rmCIRP and miRNA 130b‐3p mimic were combined 30 min prior to administration into mice intravenously.
A–FAfter 5 h of rmCIRP or miRNA 130b‐3p injection into mice, blood samples and lung tissues were collected to assess (A) AST, (B) LDH, (C) and IL‐6 in serum and mRNA for (D) IL‐6, (E) MIP‐2, and (F) KC in the lungs. AST, aspartate aminotransferase; LDH, lactate dehydrogenase; MIP‐2, macrophage inflammatory protein‐2; KC, keratinocyte chemoattractant.GLung tissue was collected after 5 h from PBS‐ and rmCIRP (5 mg/kg BW)‐treated mice with or without miRNA 130b‐3p mimic (12.5 μl of 1,000 μM) and stained with H&E. Each slide was observed under light microscopy at 400× original magnification. Representative images for each group are shown. Scale bar, 50 μm.HHistological injury scores of the lungs in different groups were quantified as described in Materials and Methods.Data information: Data are expressed as means ± SE (A–F: *n* = 5–8 mice/group; H: *n* = 15 HPF/group) and compared by one‐way ANOVA and SNK method (*P <* *0.05 vs. PBS‐treated mice; ^#^ *P *<* *0.05 vs. rmCIRP‐treated mice).

We assessed injury markers [aspartate aminotransferase (AST) and lactate dehydrogenase (LDH)] and pro‐inflammatory cytokine IL‐6 after injecting rmCIRP with or without miRNA 130b‐3p mimic into mice. We found that rmCIRP injection into mice significantly increased the serum levels of AST, LDH, and IL‐6 (Fig 5A–C). When compared to rmCIRP alone injected mice, the mice injected with rmCIRP and miRNA 130b‐3p mimic together had significantly decreased levels of AST, LDH, and IL‐6 by mean values of 25, 28, and 62%, respectively (Fig 5A–C). To confirm whether or not this miRNA mimic‐dependent inhibition of eCIRP‐induced inflammation was specific to the treatment with miRNA 130b‐3p mimic, we also injected mice with rmCIRP alone or with miRNA 140 mimic. Surprisingly, treatment with rmCIRP combined with miRNA 140 mimic did not reduce rmCIRP‐induced elevated levels of serum AST, LDH, and IL‐6 in mice (Table EV4). We also assessed the expression of IL‐6 and chemokines like macrophage inflammatory protein‐2 (MIP‐2) and keratinocyte chemoattractant (KC) in the lung tissues after administration of rmCIRP with and without miRNA 130b‐3p mimic. The expression of IL‐6, MIP‐2, and KC was upregulated following treatment of mice with rmCIRP alone (Fig 5D–F). By contrast, the co‐treatment of mice with rmCIRP and miRNA 130b‐3p mimic significantly decreased the expression of IL‐6, MIP‐2, and KC by mean values of 47, 50, and 45%, respectively, when compared to rmCIRP alone (Fig 5D–F). Thus, miRNA 130b‐3p suppresses eCIRP's pro‐inflammatory effects in mice. It has previously been proven that mice injected with rmCIRP *i.v*. develop ALI 7. The PBS‐treated mice showed normal lung architecture (Fig 5G). The rmCIRP‐injected mice developed ALI as evidenced by the presence of hyaline membranes, proteinaceous debris in the alveoli, neutrophils in the alveolar space, neutrophils in the interstitial space, and alveolar septal wall thickening (Fig 5G). There was a significantly increased lung injury score with rmCIRP alone compared to PBS‐treated mice (Fig 5H). On the other hand, mice co‐injected with rmCIRP and miRNA 130b‐3p mimic had a significantly decreased lung injury score by 28% compared to rmCIRP alone mice (Fig 5H). Therefore, treatment of miRNA 130b‐3p mimic protects mice from rmCIRP‐induced ALI.

### miRNA 130b‐3p mimic protects mice from polymicrobial sepsis

**Figure 6:**
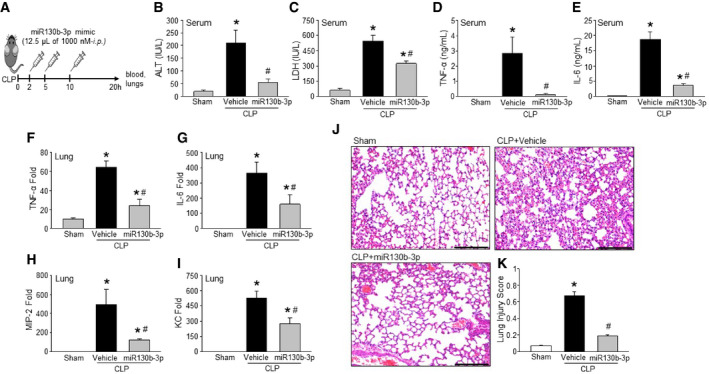
MicroRNA 130b‐3p mimic protects mice from sepsis A–ISepsis was induced in mice by CLP. Mice were injected with either miRNA 130b‐3p mimic at the concentration of 125 nM (12.5 μl of 1,000 nM miRNA 130b‐3p mimic) in 100 μl of PBS or equal volume of PBS as vehicle at 2, 5, and 10 h after CLP via intraperitoneal injection. After 20 h of CLP, blood samples and lung tissue were harvested to assess tissue injury markers (B) ALT, (C) LDH, inflammatory cytokines (D) TNF‐α and (E) IL‐6 in serum, and the expression of (F) TNF‐α, (G) IL‐6, (H) MIP‐2, and (I) KC in the lungs. ALT, alanine aminotransferase; CLP, cecal ligation and puncture; IL‐6, interleukin‐6; KC, keratinocyte chemoattractant; LDH, lactate dehydrogenase; MIP‐2, macrophage inflammatory protein‐2; TNF‐α, tumor necrosis factor‐α.JRepresentative images of H&E‐stained lung tissue at 200×. Scale bar, 100 μm.KLung injury score calculated at 200×. *n* = 5 high‐powered fields/group. Each group contains five mice. CLP, cecal ligation and puncture.Data information: Data are expressed as means ± SE (A‐I: *n* = 10 mice/group) and compared by one‐way ANOVA and SNK method (A‐I: *P <* *0.05 vs. PBS‐treated mice; ^#^ *P *<* *0.05 vs. vehicle‐treated septic mice; K: *P <* *0.05 vs. sham and ^*#*^ *P *<* *0.05 vs. vehicle mice).

We next evaluated the efficacy of miRNA 130b‐3p mimic in a clinically relevant model of sepsis in mice as induced by cecal ligation and puncture (CLP). We injected miR130b‐3p mimic at 2, 5, and 10 h after CLP via intraperitoneal injection (Fig 6A). Three consecutive doses were given to maintain adequate blood levels of miR130b‐3p mimic. We choose post‐treatment of miR130b‐3p mimic in order to provide time for eCIRP to be enriched in the serum after sepsis, allowing miR130b‐3p mimic to find its target eCIRP in the circulation. CLP caused robust increases in organ injury markers alanine aminotransferase (ALT) and LDH in the serum, while the miRNA 130b‐3p‐treated mice showed significant decrease in their levels by 73.3 and 40%, respectively (Fig 6B and C). Similarly, the serum TNF‐α and IL‐6 were elevated by CLP; however, miR130b‐3p treatment significantly reduced these levels by 96 and 80%, respectively (Fig 6D and E). Expression of the pro‐inflammatory cytokines TNF‐α, IL‐6, and chemokines like MIP‐2 and KC mRNA in lung tissues was increased in CLP‐induced sepsis and was significantly reduced with miRNA 130b‐3p mimic treatment by 63, 56, 75, and 47.5%, respectively (Fig 6F–I). Histological images of lung tissue in CLP mice displayed significant damage, with increased levels of alveolar congestion, proteinaceous debris, interstitial and alveolar neutrophil infiltration, intra‐alveolar capillary hemorrhages, and damage of epithelial architecture (Fig 6J). Treatment of CLP mice with miR130b‐3p dramatically improved these histological injury parameters in septic mice (Fig 6J). These histological changes were reflected in a significant decrease in lung tissue injury score in miR 130b‐3p‐treated mice compared to vehicle mice (Fig 6K). Therefore, administration of miR 130b‐3p mimic exhibits excellent therapeutic potential against murine polymicrobial sepsis.

### miRNA 130b‐3p mimic blocks the interaction of CIRP to TLR4/MD2 complex

**Figure 7:**
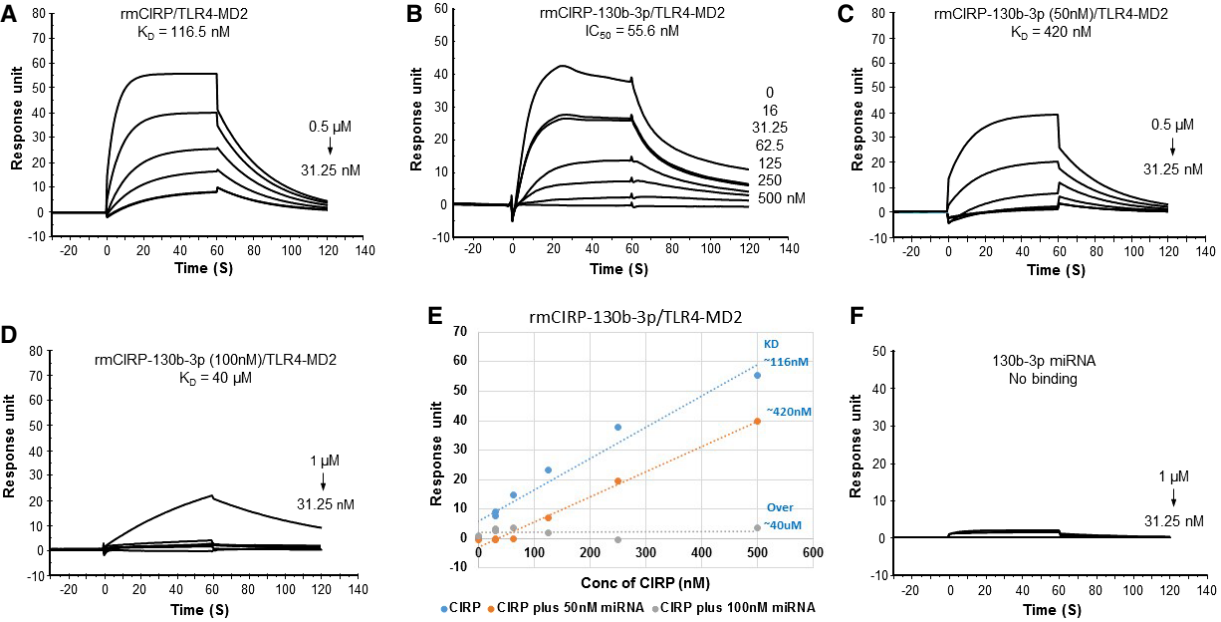
MicroRNA 130b‐3p mimic attenuates rmCIRP's binding to TLR4/MD2 complex Using surface plasmon resonance analysis, TLR4/MD2 complex was immobilized onto a CM5 series chip (GE Healthcare).
AVarying concentrations of rmCIRP (31.25–500 nM) were injected alone as the analyte (*K*_d_ of 116.5 ± 0.8 nM).BIC_50_ (55 nM) was analyzed by injecting a steady concentration of rmCIRP (0.25 μM) combined with varying concentrations of miRNA 130b‐3p (16–500 nM) over immobilized TLR4/MD2 complex.C, DVarying concentrations of rmCIRP (31.25–1,000 nM) with a constant concentration of 50 nM and 100 nM of miRNA 130b‐3p were injected as analytes over immobilized TLR4/MD2 complex (*K*_d_ of 420 nM and 40 μM, respectively).EExperiments (A, C, and D) plotted to compare response units and *K*_d_ values.FTo determine that miRNA 130b‐3p does not bind TLR4/MD2 complex directly, varying concentrations of miRNA 130b‐3p (31.25 nM up to 1,000 nM) were injected as the analyte with TLR4/MD2 complex immobilized onto a CM5 series chip. At least three independent BIAcore experiments were performed.

It has previously been shown that eCIRP promotes its pro‐inflammatory effects by binding its receptor, the TLR4/MD2 complex 8. We therefore studied the inhibitory effects of miRNA 130b‐3p mimic on rmCIRP's ability to bind to the TLR4/MD2 complex. When TLR4/MD2 complex was fixed to the chip and rmCIRP was the analyte, the *K*_d_ value between the two was 1.17 × 10^−7 ^M (Fig 7A). However, when rmCIRP was pre‐incubated with varying concentrations of miRNA 130b‐3p mimic, there was a dose‐dependent decrease in rmCIRP's binding to TLR4/MD2 complex with an IC_50_ of 5.6 × 10^−8^ M (Fig 7B). This is further proven with subsequent BIAcore tests that kept miRNA 130b‐3p mimic concentrations constant at 50 and 100 nM, while varying concentrations of rmCIRP were injected to determine *K*_d_ values. In the presence of 50 and 100 nM of miRNA 130b‐3p mimic, the *K*_d_ values of rmCIRP to TLR4/MD2 complex increased to 4.2 × 10^−7 ^M and 4.0 × 10^−5 ^M, respectively (Fig 7C–E). Increased *K*_d_ values represent decreased binding affinities. To rule out the possibility of the miRNA 130b‐3p mimic binding separately to the TLR4/MD2 complex without rmCIRP, and thereby blocking rmCIRP from its receptor, a BIAcore study was done with miRNA 130b‐3p mimic as the analyte alone with the TLR4/MD2 complex fixed to the chip. The study showed there was no binding between miRNA 130b‐3p mimic and TLR4/MD2 complex (Fig 7F). Therefore, miRNA 130b‐3p binds to rmCIRP directly and decreases its affinity for the TLR4/MD2 complex, which ultimately leads to miRNA 130b‐3p's inflammatory inhibitory effects on rmCIRP.

## Discussion

The existence of intact, cell‐free miRNAs detected in blood samples has raised several questions as to their extracellular functions. These miRNAs are preserved in circulation and are quantifiable, which supports the potential role as diagnostic or progression biomarkers in various diseases 13, 24, 25. Although the presence of various miRNAs in the blood has been identified in various diseases, their specific function in the extracellular environment is not fully understood. Our current study looked into the extracellular effect of a specific miRNA (i.e., miRNA 130b‐3p), and its interaction with a novel DAMP, eCIRP, that is known to exert pro‐inflammatory effects via its membrane receptor TLR4/MD2 complex. While our data suggest that endogenous miRNA 130b‐3p can bind to eCIRP, and the synthetic miRNA 130b‐3p mimic can inhibit the activity of eCIRP *in vivo*, it remains to be demonstrated that the mild increase in miRNA 130b‐3p seen in sera feeds back to inhibit eCIRP‐driven inflammation. Hence, further studies to determine the optimum increase in the serum levels of miRNA 130b at various time‐points of sepsis would be worthwhile to clearly establish the inhibitory effect of extracellular miRNA 130b‐3p on eCIRP‐induced inflammation.

Since there are numerous miRNAs that have already been discovered, we narrowed our scope of research by screening inflammatory relevant miRNAs using a PCR array analysis. After determining which inflammatory miRNAs had a propensity to become elevated in the blood during sepsis, we then reviewed the literature to decide which miRNAs to continue studying for their potential interaction with eCIRP. miRNAs 130b‐3p, 27b, and 140, which were each elevated on our PCR array during sepsis, have all been shown to regulate inflammation, specifically via NF‐κB 21, 22, 23, 26. Their effects are mainly executed inside the cells, still leaving the question of their roles in the extracellular spaces.

MicroRNA 130b‐3p has been described in several pathological states, some of which include inflammatory conditions, bladder cancer, hepatocellular cancer, diabetic nephropathy, lung cancer, and lupus 23, 26, 27, 28, 29, 30. Most importantly, serum levels of miRNA 130b‐3p can be measured and followed 28, 29, 31, 32. Both increased and decreased levels of miRNA 130b‐3p in blood have been associated with diseased states (i.e., hepatocellular carcinoma, nephroblastomas, and tubulointerstitial fibrosis) 31, 32, 33. The relation of inflammation and miRNA 130b‐3p has been previously described intracellularly. Its effects have been linked to the NF‐κB pathway in hepatocytes 23. However, no studies have strictly looked at the role miRNA 130b‐3p plays in the extracellular environment during inflammation. Our study provides a stepping stone into the novel mechanisms by which extracellular miRNAs exert their effects in the extracellular environment, specifically in relation to suppressing inflammation.

In the current study, we found increased contents of miRNAs 130b‐3p, 27b, and 140 in the blood of mice during sepsis. Other studies have previously found these miRNAs to be increased in the blood during other pathological conditions as well 28, 34. We recently identified a new DAMP, eCIRP, which becomes elevated in the blood during sepsis, hemorrhage, and I/R injuries [reviewed in 7]. Considering eCIRP and miRNAs 130b‐3p, 27b, and 140 are all increased in the blood during sepsis, along with the fact that CIRP is an RNA‐binding protein bearing the RNA‐binding motif, we set out to determine the interaction between eCIRP and these miRNAs in the extracellular environment. Specifically, we wanted to know whether the interaction of miRNAs with eCIRP could alter the inflammatory function of eCIRP. Our data clearly showed the binding of these miRNAs to rmCIRP with varied affinities. However, miRNA 130b‐3p had the strongest binding capability. In order to reveal the functional effects, we treated macrophages with rmCIRP alone and in combination with miRNAs 130b‐3p, 27b, or 140. Surprisingly, we noticed that among them, miRNA 130b‐3p mimic had a significant inhibitory effect on rmCIRP‐mediated macrophage production of the pro‐inflammatory cytokine TNF‐α. When these miRNAs were added alone with no rmCIRP, there was no increase in inflammatory cytokines, which shows that alone these miRNAs do not activate inflammation.

As the above studies were performed *in vitro*, we wanted to determine the *in vivo* relationship between eCIRP and extracellular miRNA 130b‐3p by carrying out a co‐immunoprecipitation experiment using serum samples obtained from septic mice. The results showed there was a significant increase in miRNA 130b‐3p levels in the co‐IP with native form of CIRP Ab vs. the co‐IP with denatured CIRP Ab, suggesting that extracellular miRNA 130b‐3p is bound to eCIRP in the blood during sepsis. Using a computational virtual modeling tool, we further revealed eCIRP's binding to extracellular miRNA 130b‐3p. Thus, our *in vitro* Biacore data, *in vivo* co‐IP (pull‐down) findings, and finally the virtual modeling unequivocally proved eCIRP's novel interaction with extracellular miRNA 130b‐3p in sepsis. eCIRP has been previously shown to cause inflammation and ALI 7. Using an *in vivo* sterile inflammation model and a pre‐clinical model of murine polymicrobial sepsis, we further deduced that the treatment of miRNA 130b‐3p mimic was also effective in mitigating systemic inflammation of eCIRP or non‐sterile model of sepsis by measuring injury markers and ALI in mice. Combined, these findings suggest that miRNA 130b‐3p is an endogenous inhibitor of eCIRP. Synthetic decoy, oligonucleotides (ODNs), and mimicking responsive elements of potent transcription factors like NF‐kB and AP‐1 were shown to attenuate inflammation 35. Since HMGB1 is a DNA‐binding protein, strategy utilizing oligos that recognize HMGB1 also attenuate inflammation 36. Nonetheless, our finding of extracellular miR130b‐3p serving as an RNA decoy molecule to attenuate eCIRP's inflammatory action is novel because of the fact that we discovered this endogenous inhibitor (extracellular miR130b‐3p) of eCIRP in an unbiased fashion by performing a hypothesis generating serum miRNA PCR array study.

To identify the mechanism by which miRNA 130b‐3p inhibited eCIRP's inflammatory effects, we focused on the interaction of eCIRP with its receptor, TLR4/MD2 complex. A series of BIAcore experiments were completed which revealed miRNA 130b‐3p mimic impeded the binding between rmCIRP and TLR4/MD2 complex, which possibly in turn leads to a decreased activation of the pro‐inflammatory mediators. At the same time, miRNA 130b‐3p mimic does not bind to TLR4/MD2 complex independently, and therefore does not exert inhibitory effects via the TLR4/MD2 pathway by itself. Although BIAcore studies helped us identify the binding abilities between miRNA 130b‐3p mimic, rmCIRP, and TLR4/MD2 complex, identification of the CIRP motif where miRNA binds has yet to be discovered. Future studies showing experimental proof of the structural motif of eCIRP bound to the TLR4/MD2 receptor with and without miRNA 130b‐3p mimic will be of great interest. Since other DAMPs have multiple receptors, eCIRP may have other unidentified receptors that can lead to activated inflammation. Our current findings may help design future studies that focus on the inhibition of eCIRP when bound to these receptors.

One possible concern when working with miRNA mimics is their stability in the extracellular space. Endogenous miRNAs are stable due to the protection of exosomes or bound proteins 14, 15. The naked miRNA mimics in the extracellular space that are not bound to a protein and not protected in an exosome will only last a few seconds in the blood before being degraded by nucleases 14, 15. Many strategies have been implored to make miRNA mimics stable in the extracellular space. Some of these methods include altering the backbone with peptides creating an achiral structure consisting of N‐(2‐aminoethyl)‐glycine units, creating a 2′‐4′ methylene bridge, replacing an oxygen with a sulfur, changing the 2′‐OH in the ribose ring to 2′‐O‐methyl, and binding mimics to a nanoparticle 37, 38. Strategies like these will most likely need to be implemented in future studies if naked miRNA mimics are planned to be injected into the extracellular space as a therapeutic inhibitor of inflammation.

Many studies focus on the intracellular function of miRNA. Therefore, the strategy to obtain intracellular miRNA in a functional state is to use double‐stranded miRNA mimics and a transfection method 19. This double‐stranded form most closely resembles miRNAs before becoming mature, allowing them to integrate into RISCs 10, 11, 19. As it incorporates itself into RISCs, it becomes single‐stranded and exerts its intracellular effects on target mRNAs 11. However, our study focused on the extracellular effects of already matured single‐stranded miRNAs in the extracellular space. Therefore, single‐stranded miRNA mimics were used in all aspects of our study.

In the present study, we determined the elevated levels of miR130b‐3p in the serum of septic mice as compared to sham mice, regardless of their potential sources or their extracellular forms. miR130b‐3p is expressed by various cell types, including epithelial cells, endothelial cells, and macrophages 39, 40, 41. Since cellular apoptosis and necrosis commonly occurs in sepsis, there is a possibility that these cells can release miR130b‐3p into the extracellular environment. Extracellular miRNAs can be found in the blood bound by membranes in exosomes and microvesicles, or bound to proteins while membrane‐free 42, 43. In the current study, we did not quantify how much of the miRNA 130b‐3p is bound by membranes vs. how much is freely circulating in the serum attached to proteins. Theoretically, extracellular miRNAs that are membrane bound will exert their effects intracellularly after merging or being engulfed by the cell. These miRNAs would not be able to interact with eCIRP and the TLR4 receptor which is on the extracellular surface of the cell membrane. However, free protein‐bound miRNAs can theoretically exert effects on cell receptors like TLR4. Future studies to determine the free vs. exosome containing miRNAs in the extracellular environment will be informative.

A recent study reported that phosphorothioate ODN can dampen inflammation driven by LPS and HMGB1 36. We excluded structural effect of the oligos by pointing to the fact that if the inhibitory effects were based on the chemistry of the backbone alone, and unrelated to the nucleotides, then all three miRNA mimics would have similar inhibitory effects on the rmCIRP‐induced inflammation. However, this was not the case and there was a clear difference in the inhibitory effects of the three miRNAs, with miRNA 130b‐3p having the strongest inhibition, while one did not have any effect. Also, miRNA 140‐5p has the same numbers of nucleotides as miRNA 130b‐3p and therefore would have had the same effects if the inhibition was related only to their backbones, since the backbones are identical. Most importantly, the manufacturer uses standard phosphodiester bonds when creating customized RNA sequencing, unless directed otherwise. Our miRNA mimics were single‐stranded RNA molecules with backbones that used phosphodiester bonds, thereby excluding phosphorothioate ODN‐dependent inhibitory effects.

In conclusion, we demonstrated a novel interaction between miRNA 130b‐3p and eCIRP, in which miRNA 130b‐3p is an endogenous inhibitor of eCIRP‐mediated inflammatory responses, via the TLR4 pathway. This may shed light on miRNA/protein interactions and lead to a new therapeutic strategy in sepsis and other inflammatory diseases.

## Materials and Methods

### Human subjects

Blood samples were obtained from de‐identified patients admitted to the Department of Emergency Medicine, North Shore University Hospital, Manhasset, New York. For the control group, blood samples were obtained from the healthy human subjects. Blood samples were centrifuged at 400 *g* for 10 min at room temperature, and serum aliquots were stored at −80°C for the assessment of miRNA 130b‐3p and eCIRP by real‐time qPCR and ELISA, respectively. Informed consent was obtained from all participants, and human subject protocols were approved by the Institutional Review Board of the North Shore University Hospital.

### Mice

Wild‐type male C57BL/6 mice between 8 and 12 weeks of age were purchased from Jackson Laboratories, Bar Harbor, ME. Mice were housed in a temperature‐controlled room with 12‐h light cycles and fed a standard laboratory chow diet. All animal experiments were conducted in accordance with the National Institutes of Health guidelines for the care and use of laboratory animals, and the protocol was approved by the Institutional Animal Care and Use Committee at the Feinstein Institutes for Medical Research.

### Sepsis model

Sepsis was induced in mice by CLP as described previously 8. Briefly, mice were anesthetized with 2% isoflurane inhalation. After placing them in the supine position, the abdomen was shaved and wiped with 70% isopropyl alcohol followed by 10% povidone‐iodine. A midline laparotomy was performed to expose the peritoneal cavity. The appendix was located and ligated 1 cm from the distal end with a 4‐0 silk suture. The blind pouch of the appendix was then punctured through and through once with a 22‐gauge needle. At the perforated sites, a small amount of stool was expressed. The abdominal incision was then closed in two layers with 4‐0 silk suture. The sham mice underwent anesthesia and a midline laparotomy only with closure. All surgical animals received a 500 μl subcutaneous injection of normal saline for resuscitation. After 20 h, mice were euthanized and blood samples and lung tissues were collected for various analyses.

### Treatment of septic mice with miRNA 130b‐3p mimic

Sepsis was induced in mice by CLP. miRNA 130b‐3p mimic was injected in mice at the concentration of 125 nM diluted in 100 μl of PBS at 2, 5, and 10 h after CLP via intraperitoneal (i.p.) injection. The sequences of murine single‐stranded miRNA mimics are shown in Table EV5. The miRNA 130b‐3p mimic was single‐stranded RNA molecules with backbones that used phosphodiester bonds synthesized by Integrated DNA Technologies (Coralville, Iowa) and purified by RNase‐free HPLC. After 20 h of CLP, we collected blood samples and lung tissue to assess various inflammatory cytokines (TNF‐α, IL‐6), chemokines (MIP‐2, KC), and organ injury markers (ALT, LDH).

### Serum miRNA PCR array and real‐time quantitative PCR (qPCR) analysis

Total miRNA was extracted from mouse and human serum using miRNeasy Serum/Plasma Kit (Qiagen, Hilden, Germany). MicroRNA isolation from the serum was controlled for with spike‐in of *Caenorhabditis elegans*. cDNA was created using miScript II RT kit (Qiagen). Reverse transcription occurs via creating a polyadenylated tail on the mature miRNAs by poly(A) polymerase with subsequent reverse transcription using oligo‐dT primers (Qiagen). We used mouse Inflammatory Response and Autoimmunity miScript miRNA PCR Array kit purchased from Qiagen (Catalog No.: MIMM‐105Z) which has primers of 84 different miRNAs that have been implicated with inflammation. PCR amplification for miRNA PCR array was performed on StepOnePlus real‐time machine (Applied Biosystems, Foster City, CA). For confirmation of the expression of selected miRNAs of mouse and human serum, we isolated total miRNA from serum and reverse‐transcribed to make cDNA following the above protocol and kits from Qiagen. qPCR amplification was performed on StepOnePlus real‐time machine (Applied Biosystems) using mouse miRNA 130b‐3p, 27b, and 140 and human miRNA 130b‐3p‐specific primers (Qiagen). *C. elegans* miRNA 39‐3p was spiked in and used to normalize comparative cycle threshold (CT) values. It is worth mentioning that human and mouse miRNA 130b‐3p sequences are the same.

### Surface plasmon resonance analysis

BIAcore T200 (GE Healthcare Bio‐Sciences, Piscataway, NJ) was used for real‐time binding interaction studies. For binding analysis of rmCIRP and miRNA biotinylated single‐stranded mimics (Sequence and Integrated DNA Technologies, Skokie, IL), the biotinylated miRNAs 130b‐3p, 27b, and 140 were individually immobilized onto separate SA (streptavidin) chips (GE Healthcare). rmCIRP was then sequentially injected at a starting concentration of 500 nM, which was serially diluted to 7.8 nM. The equilibrium dissociation constant (*K*_d_) was obtained to evaluate the binding affinity by using the BIAEvaluation 2.0 software (GE Healthcare) supposing a 1:1 binding ratio. For TLR4/MD2 complex and CIRP‐binding analyses, human TLR4/MD2 complex (R&D Systems, Cat. No. 3146‐TM‐050) was immobilized onto a CM5 series chip (GE Healthcare). To evaluate miRNA 130b‐3p's inhibition of rmCIRP binding to its receptor, TLR4/MD2 complex, rmCIRP (0.25 μM) with varying concentrations of miRNA 130b‐3p (16–500 nM) was injected over a TLR4/MD2 complex immobilized chip. Varying concentrations of rmCIRP (31.25–1,000 nM) were then incubated with either 50 or 100 nM of miRNA 130b‐3p and sequentially injected over a TLR4/MD2 complex immobilized chip to determine *K*_d_ values. The *K*_d_ values were obtained using the BIAEvaluation 2.0 software (GE Healthcare), supposing a 1:1 binding ratio. To evaluate whether there was any independent binding of miRNA 130b‐3p to TLR4/MD2 complex, miRNA 130b‐3p was sequentially injected with concentrations ranging from 31.25 nM up to 1,000 nM over a TLR4/MD2 complex immobilized chip. Binding experiments were conducted in 1XHBS‐EP as the running buffer.

### Co‐immunoprecipitation of *in vivo* eCIRP attached to miRNA 130b‐3p

Serum was obtained from mice 20 h after CLP or sham operation was performed. A constant volume of serum for each sample was incubated with either commercial CIRP Ab (Proteintech, Rosemont, IL) or denatured CIRP Ab as negative control overnight at 4°C. Denatured CIRP Ab was prepared by heating the commercial CIRP Ab at 95°C for 5 min. Using the Pierce Classic Magnetic IP/Co‐IP Kit (Thermo Fisher Scientific), magnetic immunoprecipitation was performed in order to extract eCIRP from the serum, including attached molecules to eCIRP. miRNA was then isolated from the immunoprecipitation using miRNeasy Serum/Plasma Kit (Qiagen). miScript II RT Kit from Qiagen was then used to reverse transcribe cDNA. PCR amplification was performed on StepOnePlus real‐time machine (Applied Biosystems) using miRNA 130b‐3p‐specific primers (Qiagen). Heparin was not used to collect the blood samples for the quantitative assay of miRNA 130b‐3p by qPCR. For serum, *C. elegans* miRNA 39‐3p was spiked in and used to normalize comparative CT values.

### Treatment of macrophages with miRNA 130b‐3p mimic

Mouse macrophage cell line RAW264.7 cells were purchased from American Type Culture Collection (ATCC) and cultured in Dulbecco's modified Eagle medium (DMEM; Life Technologies Corporation, Grand Island, NY) with 10% of heat‐inactivated fetal bovine serum (FBS; MP Biomedicals, Santa Ana, CA), 100 U/ml of each penicillin and streptomycin (Thermo Fisher Scientific, Waltham, MA), and 5% glutamine (Life Technologies Corporation). Prior to experiments, cells were washed with PBS and switched to OPTI‐MEM (Life Technologies Corporation) devoid of FBS for a total of 24 h. Cells were treated with 1 μg/ml of recombinant mouse (rm) CIRP with or without single‐stranded miRNA mimic 130b‐3p (Integrated DNA Technologies) at doses of 10, 100, and 1,000 nM. rmCIRP and miRNA mimic 130b‐3p were combined 30 min prior to stimulation of RAW264.7 cells. Mouse peritoneal cavity (PerC) macrophages were obtained via peritoneal lavage using 10 ml of PBS with 2% FBS. PerC macrophages were then isolated by centrifugation at 100 *g* for 10 min and plated for 24 h in a humidified incubator at 37°C with 5% CO_2_ atmosphere using RPMI (Roswell Park Memorial Institute) medium (Life Technologies Corporation) with 10% FBS. After 24 h, PerC macrophages were mechanically lifted and plated in RPMI with 1% FBS medium at a concentration of 1 × 10^6^ cells/ml. They were treated with either rmCIRP alone, or in combination of rmCIRP with various concentrations of miRNA mimic 130b‐3p, which were combined 30 min prior to administration.

### Isolation of mRNA from lungs and real‐time qPCR

mRNA was extracted from lung tissues with TRIzol reagent (Invitrogen). Equal amount (3 μg) of mRNA was reverse‐transcribed into cDNA using reverse transcriptase enzyme (Applied Biosystems). The qPCR was performed from the diluted cDNA templates with forward and reverse primers (Table EV5) and SYBR Green PCR Master Mix (Applied Biosystems) using Applied Biosystems 7300 real‐time PCR machine. Mouse β‐actin served as an internal control gene for normalization. Relative expression of mRNA was represented as fold change in comparison with the PBS‐treated group.

### Induction of inflammation by injecting mice with recombinant mouse CIRP

Mice were injected with rmCIRP (5 mg/kg BW) with either miRNA 130b‐3p or normal saline via the internal jugular vein. The rmCIRP was pre‐incubated with miRNA 130b‐3p (12.5 μl of 1,000 nM) for 30 min on ice before injection. Animals were euthanized 5 h later, and blood samples and lung tissues were collected for various analyses.

### Assessment of cytokines and organ injury markers

Serum and cell culture supernatant cytokines TNF‐α and IL‐6 were measured using mouse enzyme‐linked immunosorbent assays (ELISA) kits (BD Biosciences, San Diego, CA). Extracellular CIRP levels in the human serum were assessed by ELISA (Catalog No.: LS‐F25521, LifeSpan Biosciences, Inc, Seattle, WA). Cytokine array was performed from the mouse peritoneal macrophage culture supernatants by using Mouse Cytokine ELISA Plate Array I (Catalog No.: EA‐4005, Signosis, Santa Clara, CA). Colorimetric assays were used to measure serum levels of ALT, AST, and LDH using kits from Pointe Scientific, Canton MI.

### Histological lung evaluation

Lung tissues were collected 5 h after injection of rmCIRP or 20 h after CLP with and without treatment with miRNA mimic 130b‐3p. Sections of lung were immediately preserved in 10% formalin before permanently fixing in paraffin. Five‐micrometer sections of lung tissue were prepared and stained with hematoxylin and eosin (H&E). Light microscopy was used for the evaluation of slides at 400× magnification. Histology scoring followed guidelines from the American Thoracic Society Workshop 44. Scoring focused on the presence of hyaline membranes, the presence of proteinaceous debris in airspaces, alveolar septal wall thickening, and neutrophil infiltration of alveolar and interstitial spaces. Scores were averaged, and each group's mean was compared.

### Virtual modeling

#### Homology modeling of CIRP

We retrieved the sequence of human CIRP from the NCBI database (BAG70039.1). The model was generated using Iterative Threading ASSEmbly Refinement (I‐TASSER) server based on templates identified by threading approach to maximize percentage identity, sequence coverage, and confidence 45. The model of CIRP was generated based on templates, that is, 1X5S (solution structure of RRM domain in A18 hnRNP), 1QM9 (tandem RNA recognition motifs from polypyrimidine tract binding protein), 2KN4 (structure of the RRM domain of SC35), 3S8S (structure of the RRM domain of human SETD1A), 4C7Q (structure of the Nicotiana tabacum GR‐RBP1 RRM domain), 4PKD (U1‐70k in complex with U1 snRNA stem‐loops 1 and U1‐a RRM in complex with stem‐loop 2), and 2FY1 (A dual mode of RNA recognition by the RBMY protein).

#### Model refinement

The 3D model of CIRP was refined using GalaxyRefine 46. The refinement process is based on repetitive relaxations by short molecular dynamics simulations for mild (0.6 ps) and aggressive (0.8 ps) relaxations with 4 fs time step after structure perturbations. The refinement of models improved certain parameters, for example, an increase in Rama favored residues and decrease in poor rotamers.

#### 3D modeling of miR130b‐3p

We retrieved the sequence of human miRNA 130b‐3p (miRBase Id = hsa‐mir‐130b‐3p) from the miRBase database 47. The 3D model of miRNA 130b‐3p was generated using RNAComposer server, which is a method based on machine translation principle and operates on the RNA‐FRABASE database 48.

#### Docking studies

The docking of CIRP and miR130b‐3p was performed using NPDock 49 that combines GRAMM program to perform a rigid body global search, ranking, and scoring of best decoys using statistical potentials, clustering of best decoys, and finally, a Monte Carlo simulated annealing procedure (with protein and nucleic acid molecules treated as rigid bodies) to optimize the protein–nucleic acid interactions in the representative clusters.

#### miRNA–protein interaction studies

The analyses of miRNA–protein interactions were performed using PDBePISA 50 and KFC2 51 servers. Visualization of PPI and miRNA–protein complexes was performed using Chimera 52.

#### CIRP and miR130b‐3p interactions

The interaction interface of CIRP and miRNA 130b‐3p has a total interface area of 957.1 Å^2^, and the solvation free energy gain upon formation of interface Δ^i^ *G* is −9.8 kcal/mol. The solvation free energy of assembly dissociation Δ*G* ^diss^ is 4.0 kcal/mol. The rigid body entropy change at dissociation *T*Δ*S* ^diss^ is 10.7 kcal/mol. The interaction interface of CIRP and miRNA 130b‐3p includes Met1, Ile57, Asp58, Lys61, Met64, Asp80, Gln81, Gly83, Lys84, Arg139, Tyr142, Ala143, Ser144, Tyr153, Asp165, His170, Asn171, and Glu172 residues of CIRP and A2, G3, U4, U9, G10, A11, U12, G13, A14, A15, A16, G17, and G18 residues of miRNA 130b‐3p. Also, there are 11 hydrogen bonds in the interface of CIRP and miRNA 130b‐3p complex. The interface residues involved in hydrogen bond formation in the CIRP, and miRNA 130b‐3p complex includes Met1 with U4 (2.66 Å) and G3 (3.87 Å), Lys61 with G3 (2.94 Å) and A2 (3.81 Å), Lys84 with A14 (2.38 Å and 3.74 Å), Ser144 with G18 (3.34 Å), His170 with U12 (3.73 Å), Glu172 with U12 (3.05 Å), Asp165 with A11 (3.49Å), and Tyr142 with A16 (3.28Å). These hydrogen bonds are very important for CIRP and miRNA 130b‐3p binding.

### Statistical analysis

Data represented in the figures are expressed as mean ± SE. ANOVA was used for one‐way comparison among multiple groups, and the significance was determined by the Student–Newman–Keuls (SNK) test. The paired Student's *t*‐test was applied for two‐group comparisons. Significance was considered for *P* ≤ 0.05 between study groups. Data analyses were carried out using SigmaPlot 12.5 graphing and statistical software (Systat Software, San Jose, CA).

## Author contributions

SDG and MA designed the experiments. SDG and HJ performed all the animal and *in vitro* experiments. SDG, MA, and HW performed human studies and analyzed human data. MH and YA‐A performed BIAcore studies and BIAcore data analysis. SDG and MA analyzed the data and prepared the figures. SDG and MA wrote the manuscript. JMN, GFC, and PW reviewed and edited the manuscript. PW conceived the idea and supervised the project.

## Conflict of interest

One of the authors (PW) is an inventor of patent applications covering the fundamental concept of targeting CIRP for the treatment of inflammatory diseases, licensed by TheraSource LLC. PW is a co‐founder of TheraSource LLC. Other authors declared that they have no competing interests.

## Supporting information



Expanded View Figures PDF

Table EV1

Table EV2

Table EV3

Table EV4

Table EV5

Review Process File
