# Supplementary material for: Extracellular microRNA 130b‐3p inhibits eCIRP‐induced inflammation
Source: EMBO Rep. 2019 Nov 14;21(1):e48075. doi: 10.15252/embr.201948075 (PMC10563445; doi:10.15252/embr.201948075)
Supplement: Supplementary file 1 — Expanded View Figures PDF [file EMBR-21-e48075-s001.pdf]

Expanded View Figures

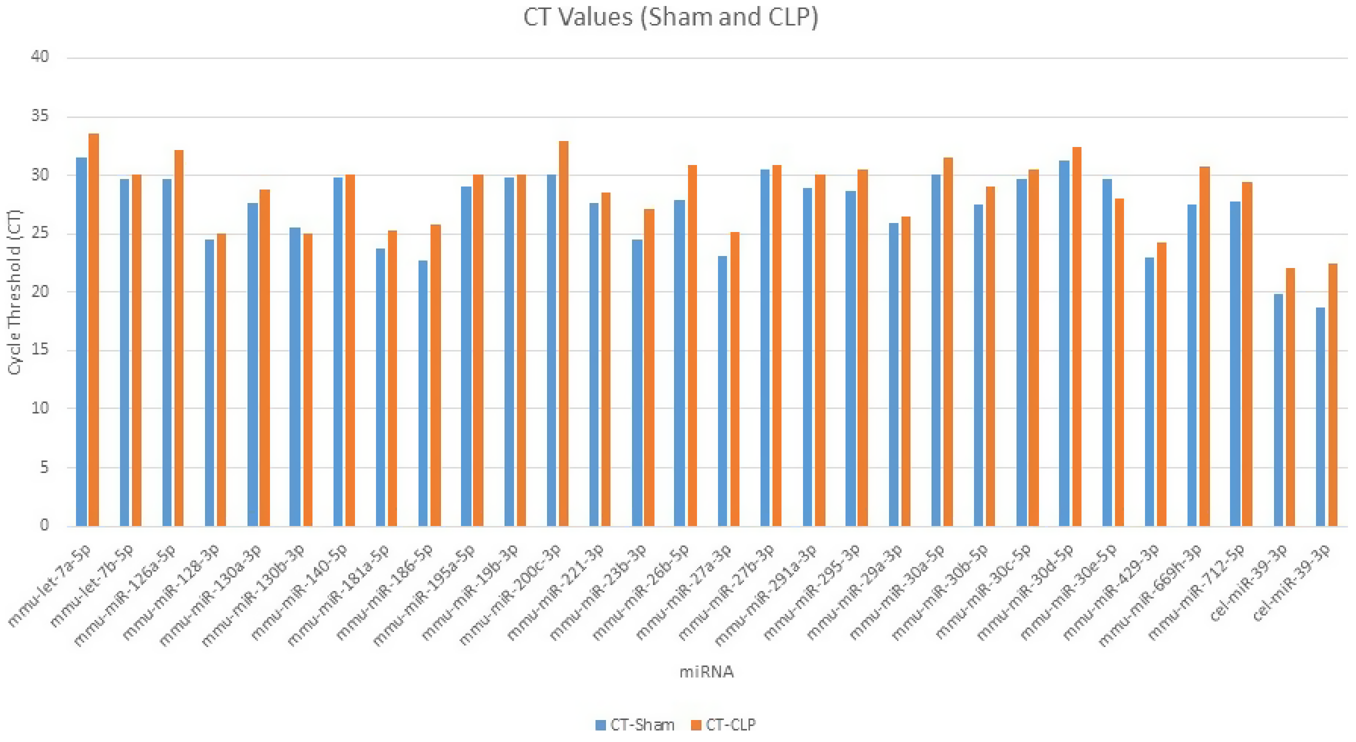

Figure EV1. Cycle threshold (CT) of miRNAs in the serum of sham and septic mice.

After 24 h of sham or CLP operation in mice, serum samples were collected and miRNAs were extracted. PCR array was performed in an Applied Biosystems StepOnePlus real-time PCR machine under the thermal profile of 95°C for 10 min and followed by 33 cycles of 95°C for 15 s and 60°C for 1 min. *Caenorhabditis elegans* miRNA 39-3p was spiked in and used to normalize comparative cycle threshold (CT) values.

 © 2019 The Authors

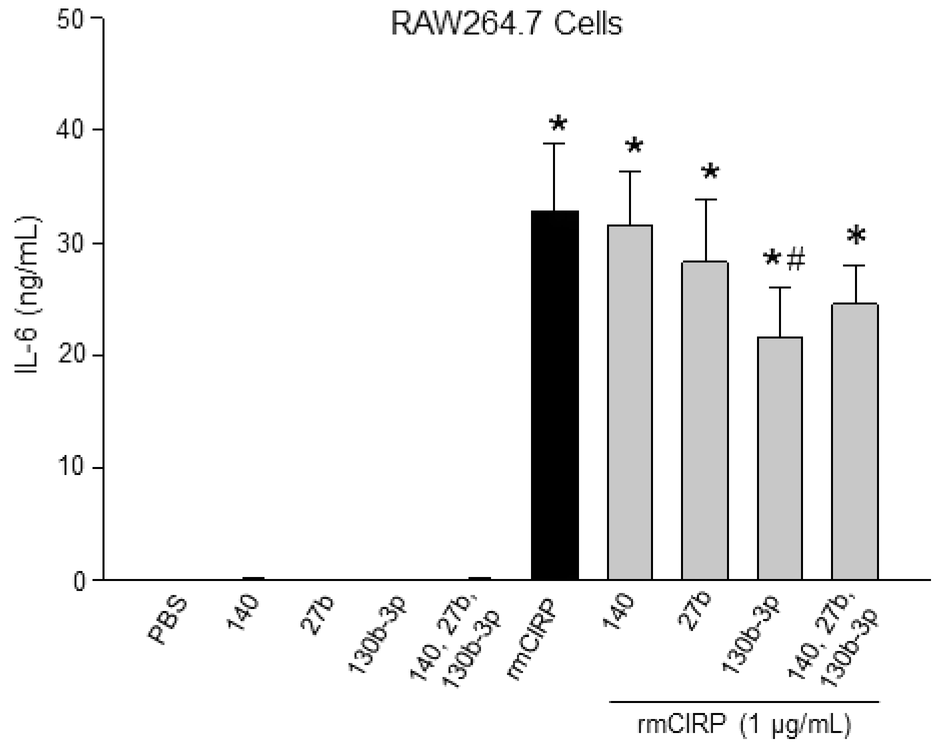

Figure EV2. MicroRNA 130b-3p attenuates eCIRP-induced IL-6 production in RAW264.7 cells *in vitro*.

RAW264.7 cells (1 × 10^6 cells/ml) were stimulated with rmCIRP (1 µg/ml) with and without each of the three miRNAs at a concentration of 100 nM or altogether with each at a concentration of 100 nM. The supernatant was collected at 24 h and assessed for IL-6 by ELISA. Data are expressed as means ± SE and compared by one-way ANOVA and SNK method (n = 6 samples/group). $^*P < 0.001$ vs. PBS. $^\#P < 0.05$ vs. rmCIRP.

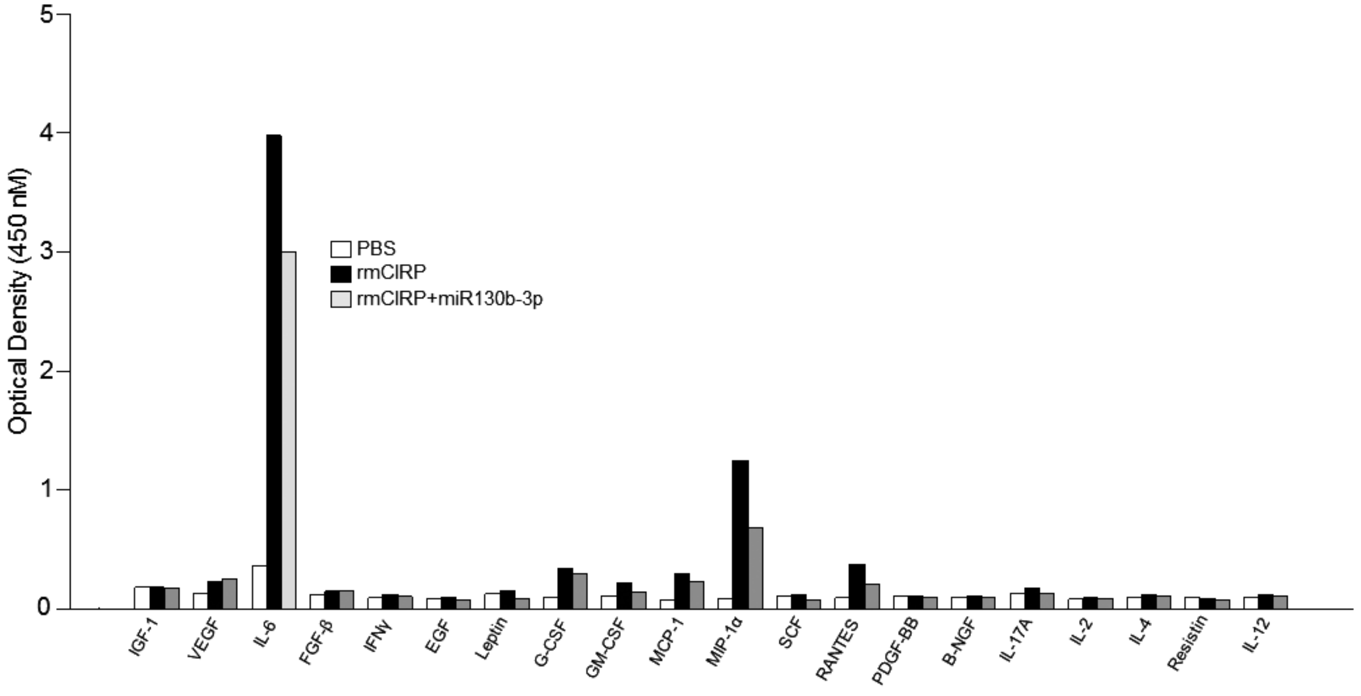

Figure EV3. Effect of miRNA 130b-3p mimic on cytokine production in rmCIRP-treated peritoneal macrophages *in vitro*.

A total of 1×10^6 cells/ml of peritoneal macrophages were treated with PBS or rmCIRP (1 μg/ml) with 100 nM of miRNA 130b-3p mimic. After 24 h of stimulation, supernatants were collected and assessed for various cytokines using Mouse Cytokine ELISA Plate Array I. rmCIRP and miRNA 130b-3p mimic were combined 30 min prior to stimulation to macrophage cells. Supernatants from $n = 3$ wells/group were pooled and performed for ELISA array. Data are expressed as optical density at 450 nM.

 © 2019 The Authors
