# Supplementary material for: Extracellular microRNA 130b‐3p inhibits eCIRP‐induced inflammation
Source: EMBO Rep. 2019 Nov 14;21(1):e48075. doi: 10.15252/embr.201948075 (PMC10563445; doi:10.15252/embr.201948075)
Supplement: Supplementary file 5 — Table EV4 [file EMBR-21-e48075-s006.docx]

**Table EV4:** **Treatment with miRNA 140 mimic has no effects on eCIRP-induced serum injury and inflammatory markers.** Mice were injected with PBS or rmCIRP (5 mg/kg BW) *i.v.* together with or without each of miRNA 130b-3p and 140 at a dose of 12.5 µl of 1000 µM. In the miRNA mimic treatment groups, rmCIRP and miRNA mimic were combined 30 min prior to administration into mice intravenously. After 5 h of rmCIRP or miRNA mimic injection into mice, blood was collected to assess AST, LDH, and IL-6 in serum.

Data information: Data are expressed as means ± SE (n=5 mice/group) and compared by one-way ANOVA and SNK method [^*^p<0.05 vs. PBS-treated mice; ^#^p<0.05 vs. rmCIRP+Vehicle (Saline)-treated mice].

| **Serum injury and inflammatory markers** | **PBS** | **rmCIRP+Vehicle** | **rmCIRP+miR130b-3p** | **rmCIRP+miR140** |
| --- | --- | --- | --- | --- |
| AST (IU/L) | 48.7 ± 7.6 | 112.44 ± 21.82^*^ | 53.8 ± 9.45^*,#^ | 105.81 ± 20.18^*^ |
| LDH (IU/L) | 50.3 ± 11.4 | 131.66 ± 24.46^*^ | 72.8 ± 7.5^*,#^ | 136.4 ± 34.7^*^ |
| IL-6 (pg/mL) | 74.14 ± 19.6 | 470.9 ± 44.26^*^ | 148.6 ± 43.84^*,#^ | 451.37 ± 46.9^*^ |
