# Supplementary material for: Extracellular microRNA 130b‐3p inhibits eCIRP‐induced inflammation
Source: EMBO Rep. 2019 Nov 14;21(1):e48075. doi: 10.15252/embr.201948075 (PMC10563445; doi:10.15252/embr.201948075)
Supplement: Supplementary file 6 — Table EV5 [file EMBR-21-e48075-s004.docx]

**Table EV5: Sequences of the murine miRNA mimics and primers.**

| miRNA 130b-3p | CAGUGCAAUGAUGAAAGGGCAU |
| --- | --- |
| miRNA 140-5p | CAGUGGUUUUACCCUAUGGUAG |
| miRNA 27b-3p | UUCACAGUGGCUAAGUUCUGC |
| TNF-α Forward | AGACCCTCACACTCAGATCATCTTC |
| TNF-α Reverse | TTG CTACGACGTGGGCTACA |
| IL-6 Forward | CCGGAGAGGAGACTTCACAG |
| IL-6 Reverse | GGAAATTGGGGTAGGAAGGA |
| KC Forward | GCTGGGATTCACCTCAAGAA |
| KC Reverse | ACAGGTGCCATCAGAGCAGT |
| MIP2 Forward | CCCTGGTTCAGAAAATCATCCA |
| MIP2 Reverse | GCTCCTCCTTTCCAGGTCAGT |
| β-actin Forward | CGTGAAAAGATGACCCAGATCA |
| β-actin Reverse | TGGTACGACCAGAGGCATACAG |
