# Supplementary material for: Extracellular microRNA 130b‐3p inhibits eCIRP‐induced inflammation
Source: EMBO Rep. 2019 Nov 14;21(1):e48075. doi: 10.15252/embr.201948075 (PMC10563445; doi:10.15252/embr.201948075)
Supplement: Supplementary file 7 — Review Process File [file EMBR-21-e48075-s007.pdf]

EMBO
reports

Extracellular microRNA 130b-3p inhibits eCIRP-induced inflammation

Steven D. Gurien, Monowar Aziz, Hui Jin, Haichao Wang, Mingzhu He, Yousef Al-Abed, Jeffrey M. Nicastro, Gene F. Coppa, Ping Wang

Review timeline:

Submission date:	12th Mar 2019
Editorial Decision:	18th Mar 2019
Revision received:	19th Apr 2019
Editorial Decision:	15th May 2019
Revision received:	14th Aug 2019
Editorial Decision:	19th Sep 2019
Revision received:	27th Sep 2019
Editorial Decision:	6th Oct 2019
Revision received:	18th Oct 2019
Accepted:	24th Oct 2019

Editor: Achim Breiling

Transaction Report:

No Peer Review Process File is available with this article, as the authors have chosen not to make the review process public.
